# Achieving Secondary Dispersion of Modified Nanoparticles by Hot-Stretching to Enhance Dielectric and Mechanical Properties of Polyarylene Ether Nitrile Composites

**DOI:** 10.3390/nano9071006

**Published:** 2019-07-12

**Authors:** Yong You, Ling Tu, Yajie Wang, Lifen Tong, Renbo Wei, Xiaobo Liu

**Affiliations:** Research Branch of Advanced Functional Materials, School of Materials and Energy, University of Electronic Science and Technology of China, Chengdu 610054, China

**Keywords:** nanocomposites, surface-functionalization, secondary dispersion, hot-stretching

## Abstract

Enhanced dielectric and mechanical properties of polyarylene ether nitrile (PEN) are obtained through secondary dispersion of polyaniline functionalized barium titanate (PANI-*f*-BT) by hot-stretching. PANI-*f*-BT nanoparticles with different PANI content are successfully prepared via in-situ aniline polymerization technology. The transmission electron microscopy (TEM), fourier transform infrared spectroscopy (FTIR), X-ray photoelectron spectroscopic instrument (XPS) and Thermogravimetric analysis (TGA) results confirm that the PANI layers uniformly enclose on the surface of BaTiO_3_ nanoparticles. These nanoparticles are used as functional fillers to compound with PEN (PEN/PANI-*f*-BT) for studying its effect on the mechanical and dielectric performance of the obtained composites. In addition, the nanocomposites are uniaxial hot-stretched by 50% and 100% at 280 °C to obtain the oriented nanocomposite films. The results exhibit that the PANI-*f*-BT nanoparticles present good compatibility and dispersion in the PEN matrix, and the hot-stretching endows the second dispersion of PANI-*f*-BT in PEN resulting in enhanced mechanical properties, crystallinity and permittivity-temperature stability of the nanocomposites. The excellent performances of the nanocomposites indicate that a new approach for preparing high-temperature-resistant dielectric films is provided.

## 1. Introduction

With the increasing requirements of modern microelectronic components, the miniaturized and flexible dielectric materials are attracting more and more attention for various applications [1,2]. However, up to now, a single component material has been unable to meet these demands. Although the traditional inorganic ceramic dielectrics are widely used owing to their high dielectric constant, their inherent characteristics of heavy weight, difficult processing and brittleness fail to meet the current practical application [3,4]. In comparison, polymeric materials have exhibited the advantages of being lightweight and flexible, but their low dielectric constant also limits their application to a great extent [5,6,7]. Therefore, combining the two-component materials is an effective way to overcome these limitations [8,9,10]. 

In recent years, polymer-based nanocomposites have proved to be an important dielectric material by virtue of the high dielectric permittivity, flexibility and excellent thermal stability for widely using as dielectrics in the electronic system [11,12,13,14,15]. It is mainly because the nanocomposites can absorb the dominants of polymer matrix and inorganic nanofillers. Nevertheless, the prerequisite for the nanocomposites demonstrating excellent properties is to realize good compatibility between nanoparticles and polymer matrix [16,17]. In general, modifying the micro-interface of the nanoparticles can greatly improve the compatibility of nanoparticles with matrix, which can also regulate the dielectric permittivity and thermal stability of composites [18,19,20]. Thus, it is important to design and fabricate surface functionalized nanoparticles on the basis of maintaining the properties of nanoparticles while improving the compatibility. Polyaniline (PANI), as a conducting polymer, has been widely used as the filler to compound with polymer matrix [21] or as the surface agent to modify the nanoparticles [22,23] by virtue of its excellent conductivity after doping.

Although surface-functionalized nanofillers can effectively improve their compatibility with the matrix enhancing the dispersion of nanofillers in the matrix, they will inevitably agglomerate at a high filler level, resulting in reduced mechanical properties and permittivity-temperature stability. Therefore, it is crucial to find a technology that can achieve secondary dispersion of fillers and improve the overall performance of composites [24]. Uniaxial hot-stretching, a method to achieve high orientation of polymer materials under the action of external force, realizes a secondary uniform dispersion of the fillers along the orientation direction. In addition, the hot-stretching technology can effectively promote the regularity of polymer molecular chains and improve their crystallinity [23,24,25,26,27,28].

In this paper, novel PANI-*functionalized*-nanoparticles of different polymer content via in-situ polymerization technology are fabricated and characterized in detail. Also, these surface functionalized nanoparticles are used as functional fillers to promote the performances of polyarylene ether nitrile (PEN). In addition, the PEN-based nanocomposites are uniaxial hot-stretched by 50% and 100% at 280 °C. The corresponding properties of the oriented nanocomposite films are investigated in detail.

## 2. Experimental

### 2.1. Materials

BaTiO_3_ (~60 nm, cubic) was bought from TPL Co., Dallas, Texas, USA. Potassium carbonate (K_2_CO_3_), 2, 6-dichlorobenzonitrile (DCBN), biphenol (BP), ammonium persulfate ((NH_4_)_2_S_2_O_8_), aniline (C_6_H_7_N), hydrochloric acid and alcohol were supplied by Chengdu KeLong chemicals, Chengdu, China. *N*-methyl-2-pyrrolodone (NMP) was bought from Chengdu Changzheng chemicals, Chengdu, China.

### 2.2. Preparation of PEN

PEN was prepared by 2, 6-dichlorobenzonitrile and biphenol in our laboratory through the previously reported method [28].

### 2.3. Preparation of PANI-f-BT Nanoparticles

**Figure 1:**
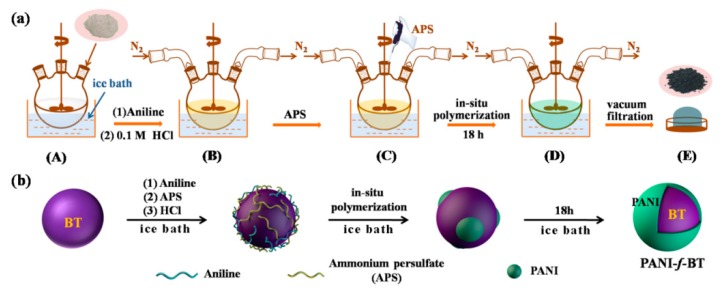
Experimental steps (**a**) and schematic diagram (**b**) of the polyaniline functionalized barium titanate (PANI-*f*-BT) nanoparticles.

The PANI-*f*-BT nanoparticles were fabricated by in-situ aniline polymerization method [29]. The specific steps are shown in Figure 1a. Firstly, BaTiO_3_ (~60 nm, 1.0 g) was added into 100 mL deionized water and ultrasonicated for 1 h. Then the dispersion was cooled to 0–5 °C with an ice bath (step A). At the same time, a certain amount of aniline dissolved in 50 mL HCl (0.1 M) was also cooled in another ice bath. Next, the aniline solution was quickly dripped into the BaTiO_3_ dispersion under nitrogen atmosphere (step B). After stirring for 1 h, pre-cooled ammonium persulfate dissolving in deionized water was added into the mixture of BaTiO_3_/aniline for oxidative polymerization for 18 h (step C and D). Finally, the PANI-*f*-BT nanoparticle was obtained through filtration and drying (step E). In addition, the preparation diagram of PANI-*f*-BT nanoparticles is shown in Figure 1b. In this system, the molar ratio of ammonium persulfate to aniline is controlled to be 1.2:1, and the amounts of aniline are 0.1, 0.2 and 0.3 mL for PANI-*f*-BT-a, PANI-*f*-BT-b and PANI-*f*-BT-c, respectively. 

### 2.4. Preparation of Nanocomposites

PEN-based nanocomposite films with 40 wt% pure BT and PANI-*f*-BT nanoparticles were prepared by solution casting method [30], which were named PEN/BT, PEN/PANI-*f*-BT-a, PEN/PANI-*f*-BT-b, PEN/PANI-*f*-BT-c, respectively.

### 2.5. Preparation of Oriented Nanocomposites by Hot-Stretching

The orientation process of nanocomposite film was carried out in a 280 °C oven by uniaxial hot-stretching method according to the previously reported paper [25]. All the nanocomposite films were stretched by 50% and 100%, respectively. The detailed steps were as follows: first of all, the two ends of the films (10 mm × 150 mm) were fixed by clamps, where the distance was controlled to be 100 mm; next, one clamp was suspended at the top of the oven, and the other one was hung with a 200 g balancing weight. The distance between the bottom of the oven and the balancing weight was adjusted to be 50 mm and 100 mm, corresponding to the stretching ratios of 50% and 100%. After the films were stretched to the required length, they were quickly removed and quenched in cold water. For comparison, the un-stretched nanocomposite films were also treated at 280 °C for the same time. 

### 2.6. Characterization

The chemical structure of PANI-*f*-BT nanoparticles was characterized on a fourier transform infrared spectroscopy (FTIR, 8400S, Shimadzu, Japan) in the transmission mode between 4000 and 500 cm^−1^ by incorporating PANI-f-BT in the KBr. The elemental analysis was tested on an X-ray photoelectron spectroscopic instrument (XPS, ESCA 2000, VG Microtech, UK) using a monochromic Al Kα (*h_v_* = 1486.6 eV) X-ray source. The micro-structures of PANI-f-BT were carried out on the transmission electron microscopy (TEM, JEM-2100F, JEOL, Japan) at 200 kV by dispersing PANI-f-BT on the copper network. The micro-structures of the PANI-f-BT nanocomposites were also obtained using a scanning electron microscopy (SEM, 6490LV, JSM, Japan) at 20 kV by sputtering gold on the fractured surface of PANI-f-BT. The crystalline structure of PANI-f-BT was characterized by X-ray diffractometer (XRD, RINT2400, Rigaku, Japan) with Cu Kα radiation. Thermal properties of samples were tested under N_2_ atmosphere by differential scanning calorimetry (DSC, Q100, TA Instruments, New Castle, USA) from 40 °C to 380 °C with a heating rate of 10 °C/min and Thermogravimetric analysis (TGA, Q50, TA Instruments, New Castle, USA) from 50 °C to 800 °C with a heating rate of 20 °C/min. The mechanical properties of the compounds were measured by Universal Testing Machine (SANS CMT6104, China) with a stretching speed of 5 mm/min. All the films were cut into standard strips (10 mm × 150 mm), and the reported data is the average value obtained by testing five samples. The Dielectric properties of the polymeric compounds were tested on a precision LCR meter (TH 2819A, Tonghui, China). The films were cut into regular pieces (10 mm × 10 mm) and both sides were coated with the conductive silver paste to form a plate capacitor.

## 3. Results and Discussion

### 3.1. Microstructure and Morphology of PANI-f-BT

In this work, enhanced dielectric and mechanical properties of PEN are obtained by hot-stretching. PANI-*f*-BT nanoparticles with different polymer content are fabricated via in-situ aniline polymerization technology by controlling the content of aniline, and then incorporated into PEN matrix by ultrasonication achieving the first dispersion of the fillers in PEN matrix. In addition, the PEN-based nanocomposites are uniaxial hot-stretched by 50% and 100% at 280 °C, obtaining the second dispersion of the fillers. Resulting from the excellent compatibility between PANI-*f*-BT and PEN and the second dispersion of PANI-*f*-BT in PEN by hot-stretching, the obtained composites demonstrate enhanced crystallinity, mechanical and dielectric properties.

**Figure 2:**
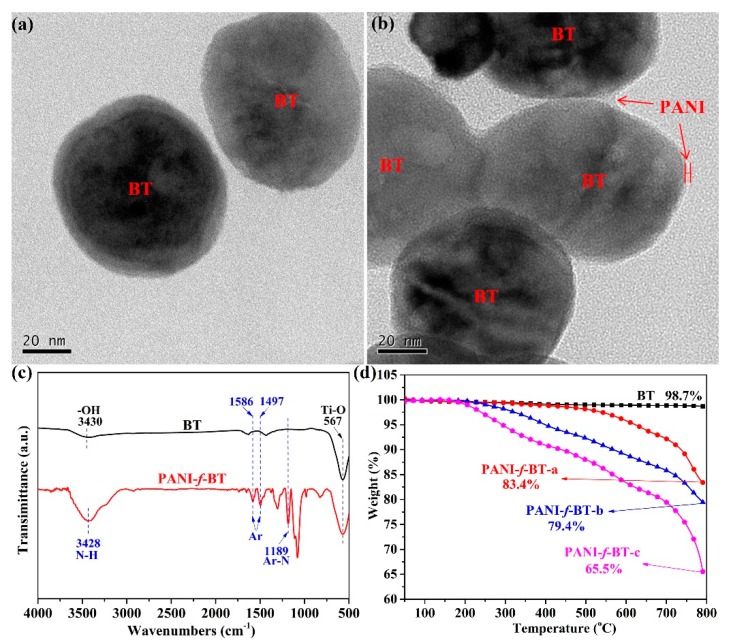
Transmission electron microscopy (TEM) images of (**a**) barium titanate (BT) and (**b**) polyaniline functionalized barium titanate (PANI-*f*-BT-b); (**c**) Fourier transform infrared spectroscopy (FTIR) spectrum of BT and PANI-*f*-BT-b; (**d**) Thermogravimetric analysis (TGA) curves of the nanofillers.

In order to characterize the microstructure of the functionalized nanoparticles, BT and PANI-*f*-BT are characterized by TEM, as shown in Figure 2a,b. It can be seen from Figure 2a that BT shows a smooth surface without distinct interface at its periphery. In comparison, Figure 2b shows different interfaces at the edges of PANI-*f*-BT. A layer of polymer corona is uniformly coated around the BT, indicating that the surface of BT is wrapped with a compact polyaniline layer [31]. The chemical structure of PANI-*f*-BT is characterized by FTIR (Figure 2c). It is clear that the strong band at 567 cm^−1^ spectra of pristine BT and PANI-*f*-BT is corresponding to the vibration of Ti-O [32]. Besides, obvious absorption bands at 1586 and 1497 cm^−1^ can be found on the FTIR spectrum of PANI-*f*-BT, which belong to the skeleton vibration of benzene rings from polyaniline [33]. Compared to BT, the additional characteristic absorption peaks at 3428 and 1189 cm^−1^ from the spectrum of PANI-*f*-BT are the absorption peaks of N-H and Ar-N vibration, which proves that polyaniline exists in the PANI-*f*-BT [33].

**Figure 3:**
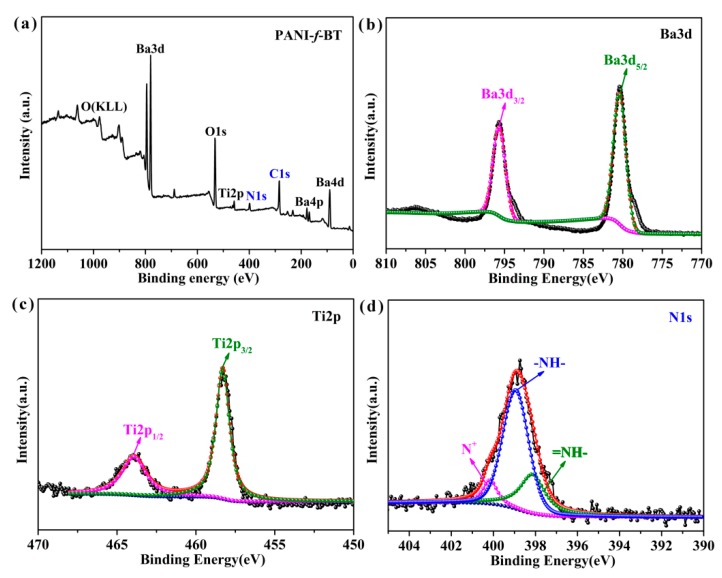
The X-ray photoelectron spectroscopic instrument (XPS) spectrum of PANI-*f*-BT-b: (**a**) full scanned spectrum; (**b**) Ba3d; (**c**) Ti2p; (**d**) N1s.

In addition, the chemical composition of the obtained PANI-*f*-BT is further characterized by XPS measurement (Figure 3). As shown in Figure 3a, it is obvious that the Ba3d, Ba4d, Ba4p, Ti2p and O1s peaks can be observed on the full scanned XPS spectrum of PANI-*f*-BT indicating the existence of BT. In addition, two peaks at 286 eV and 402 eV on the spectrum of PANI-*f*-BT are corresponding to C1s and N1s from polyaniline. Ba3d spectrum of PANI-*f*-BT presents two peaks at 779.6 and 794.9 eV which belong to Ba3d_5/2_ and Ba3d_3/2_, respectively (Figure 3b) [34]. The Ti2p spectrum of PANI-*f*-BT also shows two peaks at 457.9 eV (Ti2p_3/2_) and 463.9 eV (Ti2p_1/2_) (Figure 3c) [32]. What is more, the N1s spectrum of PANI-*f*-BT can be differentiated into three peaks: 398.1 eV (–N=), 398.9 eV (–NH–) and 400.1 eV (N^+^), respectively [35] as shown in Figure 3d.

Beside the characterization of PANI-*f*-BT, the contents of polyaniline in PANI-*f*-BT are determined by TGA test. As shown in Figure 2d, it is clear that BT nanoparticle does not demonstrate any weight loss, even when heated to 800 °C. In comparison, the residue of PANI-*f*-BT-a, PANI-*f*-BT-b and PANI-*f*-BT-c is 83.4%, 79.4% and 65.5% at 800 °C, respectively. The decrement of the residue indicates the existence of PANI in PANI-*f*-BT. Simultaneously, the less residue of the PANI-*f*-BT nanoparticles, the more PANI in the PANI-*f*-BT nanoparticles. Therefore, all these results including TEM, FTIR, XPS and TGA suggest that PANI is successfully grown on the surface of BT after the in-situ polymerization procedure.

### 3.2. Morphology of PEN/PANI-f-BT Composites

**Figure 4:**
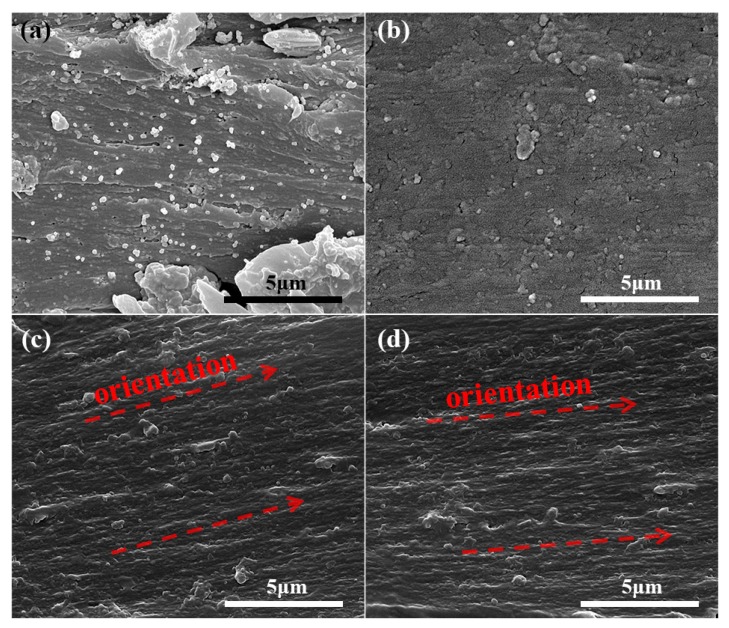
Cross-sectional scanning electron microscopy (SEM) images of (**a**) PEN/BT; (**b**) PEN/PANI-*f*-BT-b; (**c**) PEN/PANI-*f*-BT-b hot-stretched by 50%; (**d**) PEN/PANI-*f*-BT-b hot-stretched by 100%.

After the fabrication and characterization of the PANI-*f*-BT nanoparticles, they are introduced into the PEN matrix to prepare the PEN/PANI-*f*-BT composites. The miscibility between PANI-*f*-BT and PEN matrix, which is one of the most important factors affecting the properties of the nanocomposites, is firstly investigated by SEM measurement. Figure 4a shows the microstructures of PEN/BT nanocomposites, which exhibit a poor interfacial adhesion. In addition, a large number of BT nanoparticles are observed at the cross-section of the PEN matrix with serious spherical agglomeration. This phenomenon is mainly due to the high content of BT as well as the poor miscibility between PEN and BT [36]. Contrarily, the PANI-*f*-BT nanoparticles reveal a homogeneous dispersion in PEN matrix without agglomeration (Figure 4b). This result is mainly caused by that the PANI layer on PANI-*f*-BT nanoparticles which improves compatibility with PEN. Therefore, the modification of BT with PANI can effectively improve the compatibility between BT and PEN [36].

**Figure 5:**
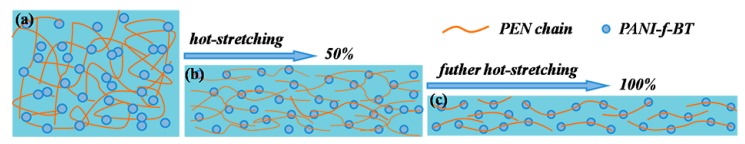
The theoretical model of the evolution process of inner network during uniaxial stretching: (**a**) the original composite film, the composite film hot-stretched by (**b**) 50% and (**c**) 100%.

To achieve the secondary dispersion of PANI-*f*-BT in PEN, the PEN/PANI-*f*-BT composites are hot-stretched in a home-made oven. Figure 4c,d are the cross-sectional SEM images of PEN/PANI-*f*-BT-b after hot-stretching at a stretching ratio of 50% and 100%, from which obvious orientation of the sample caused by the directional arrangement of polymer molecular chains under the action of external forces is observed. After hot-stretching, the PANI-*f*-BT nanoparticles are secondarily dispersed in the PEN matrix along the orientation direction. A schematic model of the evolution process of PANI-*f*-BT in the polymer matrix during uniaxial stretching is presented in Figure 5. Before the hot-stretching, the spherical PANI-*f*-BT is isotropically dispersed in the PEN matrix. With the commencement of the hot-stretching, the PANI-*f*-BT nanoparticles rearrange along the orientation direction of the stretching. Finally, the enhanced dispersion of PANI-*f*-BT in PEN matrix is obtained after the hot-stretching. Combining the improved compatibility between PANI-*f*-BT and PEN and the secondarily dispersion of PANI-*f*-BT in PEN matrix induced by hot-stretching, enhanced properties of the PEN/PANI-*f*-BT composites can be imaged.

### 3.3. Thermal Properties and Crystallization of PEN/PANI-f-BT Composites

**Figure 6:**
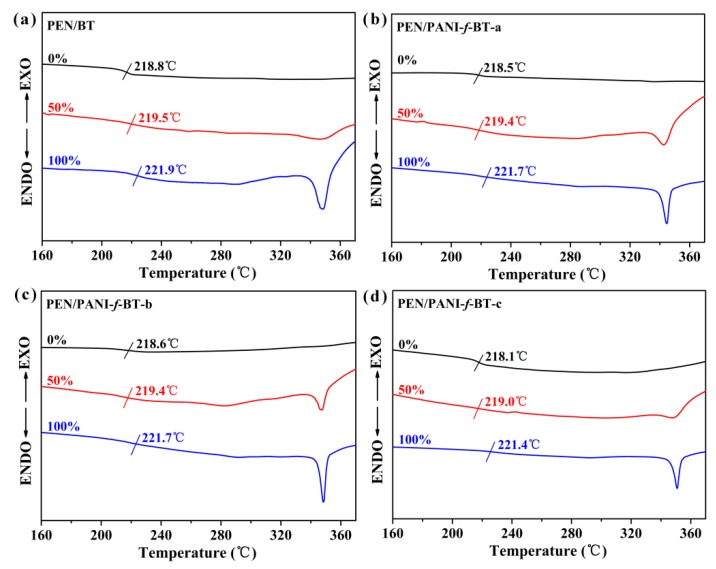
The differential scanning calorimetry (DSC) curves of nanocomposites with different stretching ratios: (**a**) PEN/BT; (**b**) PEN/PANI-*f*-BT-a; (**c**) PEN/PANI-*f*-BT-b; (**d**) PEN/PANI-*f*-BT-c.

As a crystalline polymer, the crystallization behavior of PEN is another important factor affecting the properties of the nanocomposites. The crystallization behavior of PEN/PANI-*f*-BT composites is studied by DSC and XRD. Figure 6 shows the DSC curves of the PEN/BT (Figure 6a) and PEN/PANI-*f*-BT (Figure 6b–d) nanocomposites before and after hot-stretching. It clearly shows that the melting peaks of all nanocomposites are not observed before hot-stretching, while they appear after hot-stretching. The melting enthalpy (Δ*H*_m_) of PEN/PANI-*f*-BT-b at the stretching ratios of 0%, 50% and 100% is 0, 6.4 and 9.1 J/g, respectively (Table 1). With the increase of stretching ratios from 50% to 100%, the Δ*H*_m_ of these composites increases gradually, meaning that the crystallinity of the nanocomposites increases [25,37]. This would be due to the rearrangement of PEN molecular chains during the hot-stretching resulting the transitions of the samples from amorphous regions to crystalline regions and from irregular crystals to regular crystals [28]. Besides, the half peak width of the melting peaks also shows the crystalline information of the samples. Generally, the perfect crystals exhibit smaller half peak width than the imperfect crystals. The half peak width is 9.34, 6.55, 5.68 and 5.71 °C for PEN/BT, PEN/PANI-*f*-BT-a, PEN/PANI-*f*-BT-b and PEN/PANI-*f*-BT-c respectively, at the stretching ratio of 100%. The widest half peak width of PEN/BT at 100% stretching ratio is mainly owing to the worst compatibility and dispersion of BT in the PEN matrix as confirmed by the SEM observation (Figure 4a). In addition, DSC curves also demonstrate that the glass transition (*T*_g_) of the all nanocomposites increases slightly during the hot-stretching process. For instance, the *T*_g_ of PEN/PANI-*f*-BT-b increases from 218.6 °C to 221.7 °C as stretching ratios increases from 0% to 100% (Figure 6c and Table 1). This result can also be explained by the arrangement of the PEN chains after hot-stretching, leading to the harder movement of them. Moreover, the increase of crystallinity further limits the movement of the chain segments [38]. Furthermore, the DSC curves for both cooling and heating are shown in Figure S1, as can be seen from the figure that during the first cooling scan and the second heating scan, the *T*_g_s obtained from the curves are lower than the one obtained from the first heating scan due to the supercooling effect [39]. What is more, the melting point disappears during the first cooling scan and the second heating scan due to the semi-crystalline property of the polymers and slow crystalline rate of the polymers [40]. In addition, we also characterize the crystal structure state of the samples after treatment at 200 °C, which is shown in Figure S2. It can be clearly seen from the figure that the PEN/PANI-*f*-BT-b nanocomposites with a 50% and 100% stretching ratio still show an obvious melting peak in the second heating curve after treating them at 200 °C for 10 min. It is indicated that the crystal structure of the PEN/PANI-*f*-BT-b nanocomposites is thermodynamic stable when used at 200 °C. More importantly, as shown in Figure S2d, it is clear that the melting peak is also maintained in the second heating curve of the PEN/PANI-*f*-BT-b nanocomposites with a 100% stretching ratio after treating at 300 °C for 10 min. Therefore, all these results confirm that the crystal structure of the hot-stretched PEN/PANI-*f*-BT nanocomposite films can still maintain good thermal stability during the practical application (<200 °C).

**Figure 7:**
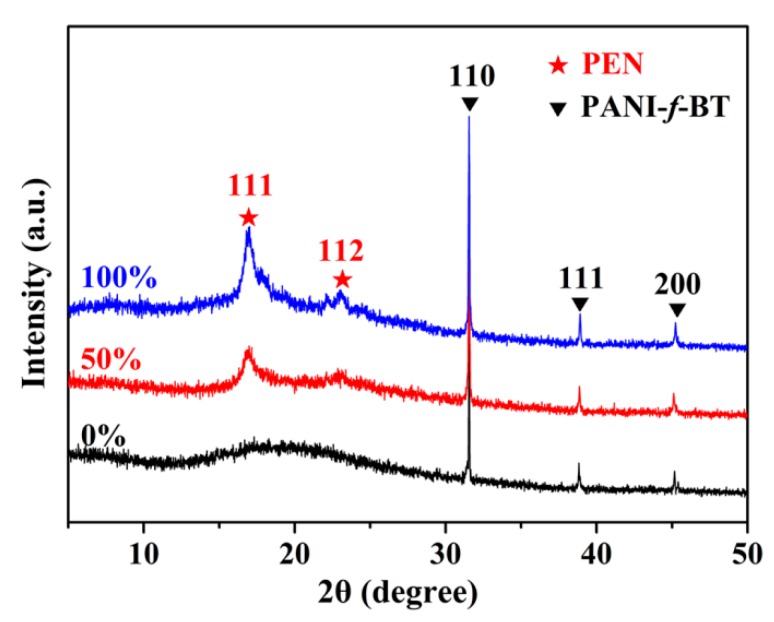
The wide-angle XRD patterns of PEN/PANI-*f*-BT-b at different stretching ratios.

XRD is usually employed to study the crystals and crystallinity of samples. Figure 7 typically shows the XRD patterns of PEN/PANI-*f*-BT-b at different stretching ratios. As shown in the figure, the diffraction peaks at around 32°, 38° and 45° are observed from all three samples which are coming from the diffractions of (110), (111) and (200) of BT. As for PEN, no crystalline peak is observed before hot-stretching. In comparison, two diffraction peaks at 17° and 23° which are coming from the diffractions of (111) and (112) of PEN are observed after the hot-stretching. The crystallinities of PEN/PANI-*f*-BT-b with the stretching ratios from 0% to 100% are 0%, 11.2% and 16.4%, which are calculated from wide-angle XRD spectrogram by using Jade 6 software [41].

### 3.4. Mechanical Properties of PEN/PANI-f-BT Composites

**Figure 8:**
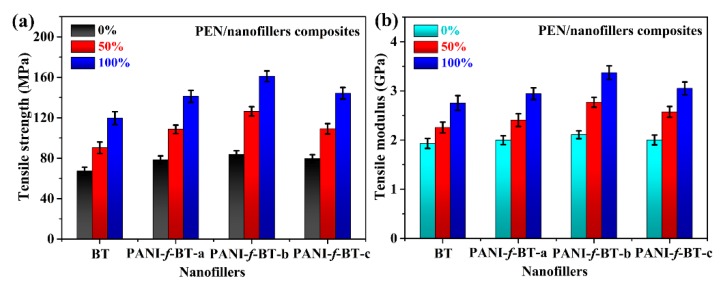
The mechanical properties of nanocomposite films at different stretching ratios: (**a**) tensile strength and (**b**) tensile modulus.

Resulting from the improved compatibility and crystalline of the composite, their enhanced properties are further investigated. Tensile strength and tensile modulus, two most important mechanical properties of the high-performance engineering plastics, are typically studied. The tensile properties of the PEN-based nanocomposites at different stretching ratios are shown in Figure 8. The tensile strength of PEN/BT nanocomposites is 67.2, 90.3 and 119.7 MPa, at the stretching ratio 0%, 50% and 100%, respectively. As expected, the tensile strengths of all nanocomposites demonstrate a significant increase after modifying the surface of BT and hot-stretching, which are shown in Figure 8a. Moreover, tensile strength of PEN/PANI-*f*-BT-b nanocomposite is 83.8 MPa without stretching. What is more, it increases to 161.1 MPa when the stretching ratio is 100%, with an increment of 90%. The detailed mechanical data of the nanocomposites are listed in Table 1. It is obvious that the tensile strengths of PEN/PANI-*f*-BT nanocomposites are higher than that of PEN/BT nanocomposites, which are attributed to the better dispersion and compatibility between PEN and PANI-*f*-BT. In addition, it can be concluded that a substantial increase in mechanical property of the nanocomposites is contributed by the high orientation of the molecular chains and the newly formed oriented crystals after hot-stretching process [25,41]. The tensile modulus of the nanocomposites exhibits a similar tendency as that of tensile strength, which results from the same reasons as mentioned above [25].

### 3.5. Dielectric Properties of PEN/PANI-f-BT Composites

**Figure 9:**
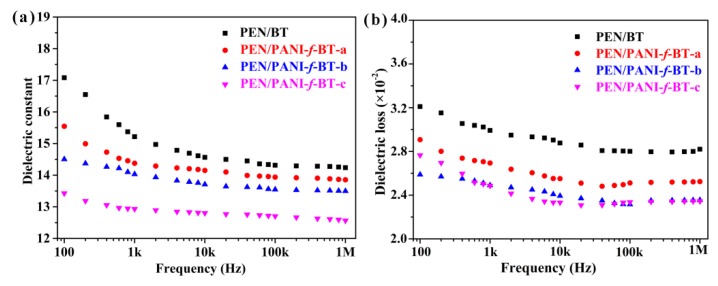
(**a**) Dielectric constant and (**b**) dielectric loss of the nanocomposites with the changing of frequency.

PEN has shown prospective application in film capacitors and other electronic devices. However, the permittivity of PEN is relatively low (~4.0 at 1 kHz) which failed to meet the high permittivity requirement for film capacitors. Herein, PANI-*f*-BT nanoparticles are used as a filler to mix with PEN for preparing PEN/PANI-*f*-BT nanocomposites. The dependence between dielectric properties of the obtained nanocomposites and varying frequency (100 Hz to 1 MHz) is shown in Figure 9a. Compared with PEN/BT, the dielectric constants of all PEN/PANI-*f*-BT nanocomposites are slightly lower than those of PEN/BT. The decreasing of dielectric constant of PEN/PANI-*f*-BT nanocomposites is caused by the PANI layer on the surface of BT nanoparticles which will hinder the charge movements from BT to PEN matrix. This usually leads to a decrease in interfacial polarization of the system [42]. In addition, it is clear that the permittivity of PEN/PANI-*f*-BT nanocomposites is more stable than that of PEN/BT nanocomposite with the change of frequency (100 Hz to 1 MHz). This is due to that the introduction of organic shell layer which enhances the compatibility between nanofillers and PEN matrix which depresses the Maxwell-Wagner polarization [30]. The dielectric loss of the PEN/PANI-*f*-BT nanocomposites is shown in Figure 9b. Although the content of nanofillers is up to 40 wt%, the dielectric loss of PEN/PANI-*f*-BT nanocomposites is still below 0.028 (1 kHz). This phenomenon also resulted from the improved compatibility between PANI-*f*-BT and PEN [30]. What is more, the dielectric loss of the PEN/PANI-*f*-BT nanocomposites demonstrates a similar trend with the changing of frequency and fillers as that of their permittivity. Furthermore, the electrical conductivity of the studied samples is shown in Figure S3. It can be seen from the figure that the electrical conductivity of PEN/PANI-*f*-BT nanocomposites is almost the same (10^−10^ S cm^−1^) at 100 Hz (Figure S3a), which indicates that all the nanocomposite films are insulators.

**Figure 10:**
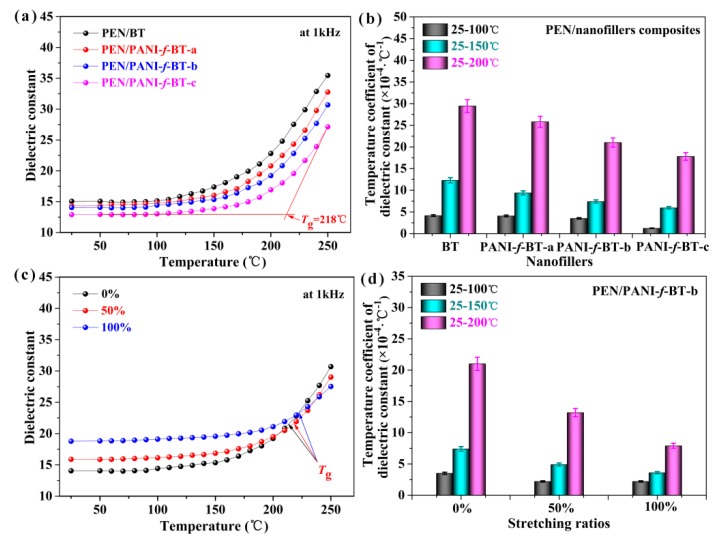
(**a**) Permittivity-temperature dependences and (**b**) temperature coefficients of dielectric constant of the nanocomposites; (**c**) permittivity-temperature dependences and (**d**) temperature coefficients of dielectric constant of PEN/PANI-*f*-BT-b at different stretching ratios.

Compared with the commonly used biaxially oriented polypropylene (BOPP) and Poly(vinylidene fluoride) (PVDF), PEN, which is a kind of special thermoplastic engineering material, has demonstrated its potential application as high temperature dielectrics. Therefore, the permittivity of all samples at different temperature is further researched. As shown in Figure 10a, the dielectric constant of nanocomposites is measured at 1 kHz in the range of 25 to 250 °C. It is clear that the dielectric constant of all nanocomposites is stable before their *T*_g_, and it increases hastily and obviously when the temperature exceeds their *T*_g_. This is due to the molecular chains of the PEN that are frozen at a temperature lower than its *T*_g_. However, as the temperature increases, the macromolecular chains are thawed which strengthens the mobility of electrons and enhances polarization in the system [43]. As a result, the *T*_g_ of the polymeric dielectrics can be obtained from their permittivity-temperature curves. As shown in Figure 10a, the *T*_g_ of PEN/PANI-*f*-BT-c obtained from the permittivity-temperature curve is 218 °C which is the same as that obtained from its DSC curve. The *T*_g_ of the other composites is also around 218 °C. The high *T*_g_ of the composites ensures the potential application of them at high temperature. Beside the qualitative description of the stable permittivity of the composites before their *T*_g_, the quantitative value of dielectric constant with the change of temperature (temperature coefficients of dielectric constant) is calculated according to Equation (1) [44]:

(1) $$\tau_{\varepsilon }=\frac{\varepsilon_{T2}-\varepsilon_{T1}}{\varepsilon_{T0}(T_{2}-T_{1})}$$

where *τ*_ε_ is the temperature coefficient of dielectric constant, *ε*_T0_ is the dielectric constant of room temperature, *ε*_T1_ is the dielectric constant of initial temperature and *ε*_T2_ is the dielectric constant of the final temperature. *T*_1_ and *T*_2_ are the initial and final temperatures, respectively. According to Equation (1), the calculated results of the temperature coefficients of dielectric constant of all nanocomposites are shown in Figure 10b. The temperature coefficients of dielectric constant of the composites are lower than 5 × 10^−4^ °C^−1^ within the temperature range from 25 to 100 °C. They are still lower than 3 × 10^−3^ °C^−1^ even though in the temperature range from 25 to 200 °C, indicating that they are extraordinarily stable, even at temperature up to 200 °C. In addition, it is notable that the temperature coefficients of dielectric constant of the composites decrease with the increase of the PANI content in the PANI-*f*-BT. This result is also largely due to the introduction of organic shell layer can improve the compatibility between nanoparticles and PEN matrix, which can effectively reduce the charge accumulation and interfacial polarization between inorganic nanoparticles and matrix [30].

Moreover, to make a clear view of the properties of the PEN/PANI-*f*-BT nanocomposites, a comparison of dielectric constant, dielectric loss and working temperature at room temperature and 1 kHz of reported polymer-based composites are summarized in Table 2. As can be seen from the table, the dielectric constant and loss of PEN/PANI-*f*-BT are 14 and 0.025 when the content of PANI-*f*-BT nanoparticles is 40 wt%. Although the dielectric constant is lower than that of the most widely used PVDF based composites, it is comparable to the P(VDF-HFP)/BT-OPA, PES/BT-CuPc and PAEN/BT@CPAEN system. In addition, the dielectric loss of PEN/PANI-*f*-BT nanocomposites maintains at a relatively low level and is lower than that of most PVDF based composites. More importantly, the inherent low temperature resistance of PVDF (<120 °C) will limit its application in high temperature environment. In comparison, in this work, the PEN/PANI-*f*-BT nanocomposites can be used as flexible dielectric films at around 200 °C, which provides a new approach for preparing high-temperature-resistant dielectric films.

The dielectric properties of the PEN/PANI-*f*-BT nanocomposites are further improved by hot-stretching. As shown in Figure 10c, the dielectric constant of PEN/PANI-*f*-BT-b is 14.0 at 1 kHz without stretching (at 25 °C). After hot-stretching, its dielectric constant increases to 15.9 and 18.7 when the stretching ratio is 50% and 100% respectively (at 25 °C). It is well-known that the micro-capacitor networks are often formed in the nanocomposites [28]. During hot-stretching, the disordered nanoparticles are realigned to form the oriented micro-capacitors in the polymer matrix along the orientation direction (Figure 5), contributing to the enhancement of the dielectric constant of nanocomposites. In addition, the electrical conductivity of PEN/PANI-*f*-BT-b nanocomposites presents a slight increase with the increase of stretching ratio (Figure S3b). This is a result of the second dispersion of PANI-*f*-BT-b in the system. What is more, the permittivity of the PEN/PANI-*f*-BT-b nanocomposites after hot-stretching at different temperatures is also investigated in this work, as shown in Figure 10c. It can be seen that the *T*_g_ of PEN/PANI-*f*-BT-b obtained from the permittivity-temperature curve increases to 219 °C and 221°C at the 50% and 100% respectively, indicating that the composites can be used at higher temperature after hot-stretching. It is well-known that the service temperature of BOPP is higher than PP due to the stretching of the sample. Furthermore, the temperature coefficients of dielectric constant of PEN/PANI-*f*-BT-b nanocomposites at different stretching ratios are shown in Figure 10d. As can be seen, the temperature coefficients of dielectric constant of PEN/PANI-*f*-BT-b nanocomposites exhibit a gradual downward trend. More importantly, the temperature coefficients of dielectric constant of PEN/PANI-*f*-BT-b are lower than 5 × 10^−4^ °C, which are very important for its application at high temperature. This is because the high orientation of the molecular chain and the increase of crystallinity limit the movement of the chain segments, which is consistent with the conclusion of the thermal properties [52]. The results reveal that the modification of the filler and hot-stretching method can effectively increase the dielectric constant and the stability of the nanocomposites at different temperatures, which presents great potential for it to be used as a high performance dielectric film in harsh environments.

## 4. Conclusions

In conclusion, enhanced dielectric and mechanical properties of polyarylene ether nitrile are obtained through secondary dispersion of polyaniline functionalized barium titanate (PANI-*f*-BT) by hot-stretching. PANI-*f*-BT nanoparticles with different PANI contents are prepared via in-situ aniline polymerization technology, and then characterized by TEM, XPS, FTIR and TGA. The results confirm that the polymer layers have uniformly enclosed on the surface of BaTiO_3_ nanoparticles. The obtained PANI-*f*-BT nanoparticles are used as functional fillers to compound with PEN for preparing the PEN/PANI-*f*-BT nanocomposites. In addition, these PEN-based nanocomposites are uniaxial hot-stretched by 50% and 100% at 280 °C to obtain the oriented nanocomposite films. The results show that the PANI-*f*-BT nanoparticles present well compatibility and dispersion in the PEN matrix, and the hot-stretching can achieve a second dispersion of the PANI-*f*-BT nanoparticles in PEN, which can effectively enhance the comprehensive properties of nanocomposites like mechanical properties, crystallinity, dielectric constant and so on. When the stretching ratios increase from 0% to 100%, the tensile strengths of PEN/PANI-*f*-BT-b nanocomposite film increase from 83.8 to 161.1 MPa, and the crystallinities increase from 0% to 16.4%. Most importantly, the permittivity temperature dependences of nanocomposites after hot-stretching are more stable than that of original nanocomposites. The excellent performances of the stretched composites indicate that these samples present a great potential to be used as a high-performance dielectric film in high-temperature environments.

## Figures and Tables

**Table 1:** Thermal and mechanical properties of nanocomposites at different stretching ratios.

Samples	*T*_g_ (°C)	Δ*H*_m_ (J/g)	Tensile Strength (MPa)	Tensile Modulus (GPa)
PEN/BT 0% 0%	218.8	-	67.2 ± 3.9	1.93 ± 0.10
PEN/BT 50%	219.5	4.2	90.3 ± 5.7	2.25 ± 0.11
PEN/BT 100%	221.9	7.5	119.7 ± 6.3	2.75 ± 0.15
PEN/PANI-*f*-BT-a 0% 0%	218.5	-	78.5 ± 3.8	1.99 ± 0.09
PEN/PANI-*f*-BT-a 50%	219.4	5.9	108.6 ± 4.2	2.41 ± 0.13
PEN/PANI-*f*-BT-a 100%	221.7	8.4	141.2 ± 5.9	2.94 ± 0.12
PEN/PANI-*f*-BT-b 0% 0%	218.6	-	83.8 ± 3.6	2.11 ± 0.08
PEN/PANI-*f*-BT-b 50%	219.4	6.4	126.4 ± 4.6	2.77 ± 0.10
PEN/PANI-*f*-BT-b 100%	221.7	9.1	161.1 ± 5.3	3.37 ± 0.14
PEN/PANI-*f*-BT-c 0% 0%	218.1	-	79.6 ± 3.9	2.01 ± 0.10
PEN/PANI-*f*-BT-c 50% 50%	219.0	4.7	109.1 ± 5.1	2.57 ± 0.11
PEN/PANI-*f*-BT-c 100% 100%	221.4	7.6	144.3 ± 5.7	3.05 ± 0.13

**Table 2:** Dielectric constant at 1 kHz and 25 °C, dielectric loss at 1 kHz and 25 °C, working temperature of typical polymer-based composites.

Samples	Content	Dielectric Constant (1 kHz, 25 °C)	Dielectric Loss (1 kHz, 25 °C)	Working Temperature (°C)	Ref.
PVDF/BT	60 vol%	95	~0.04	<120	[45]
PVDF/BT-PDOPA	50 vol%	56.8	0.04	<120	[46]
PVDF/BT-TDPA	40 vol%	48	0.03	<120	[47]
PVDF/BT-SiO_2_	10 vol%	14.7	0.02	<120	[48]
hydantoin/BT-P(VDF-HFP)	50 vol%	48.9	0.06	120	[49]
P(VDF-HFP)/BT-OPA	30 vol%	~15	0.08	120	[50]
PES/BT-CuPc	40 vol%	~17	~0.12	~160	[51]
PAEN/BT@CPAEN	40 wt%	13	0.023	~180	[30]
PEN/PANI-*f*-BT	40 wt%	14	0.025	~200	This work

## Supplementary Materials

The following are available online at https://www.mdpi.com/2079-4991/9/7/1006/s1, Figure S1: The DSC curves of PEN/PANI-*f*-BT nanocomposite films with different stretching ratios. Figure S2: The DSC curves of PEN/PANI-*f*-BT-b nanocomposite films with different stretching ratios. Figure S3: The electrical conductivity of PEN/PANI-*f*-BT nanocomposites.
