# Supplementary material for: Achieving Secondary Dispersion of Modified Nanoparticles by Hot-Stretching to Enhance Dielectric and Mechanical Properties of Polyarylene Ether Nitrile Composites
Source: Nanomaterials (Basel). 2019 Jul 12;9(7):1006. doi: 10.3390/nano9071006 (PMC6669864; doi:10.3390/nano9071006)
Supplement: Supplementary file 1 [file nanomaterials-09-01006-s001.pdf]

Supplementary Information

Achieving Secondary Dispersion of Modified Nanoparticles by Hot-Stretching to Enhance Dielectric and Mechanical Properties of Polyarylene Ether Nitrile Composites

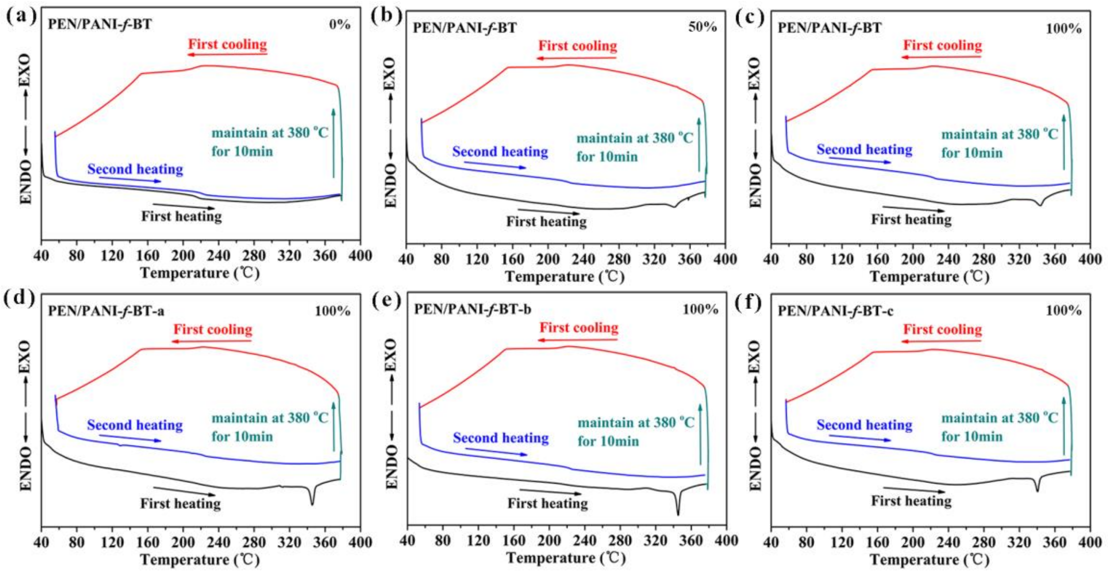

Figure S1. The DSC curves of PEN/PANI-*f*-BT nanocomposite films with different stretching ratios: (**a**) PEN/PANI-*f*-BT nanocomposites; (**b**) PEN/PANI-*f*-BT nanocomposites hot-stretched by 50%; (**c**) PEN/PANI-*f*-BT nanocomposites hot-stretched by 100%; (**d**) PEN/PANI-*f*-BT-a nanocomposites hot-stretched by 100%; (**e**) PEN/PANI-*f*-BT-b nanocomposites hot-stretched by 100%; (**f**) PEN/PANI-*f*-BT-c nanocomposites hot-stretched by 100%.

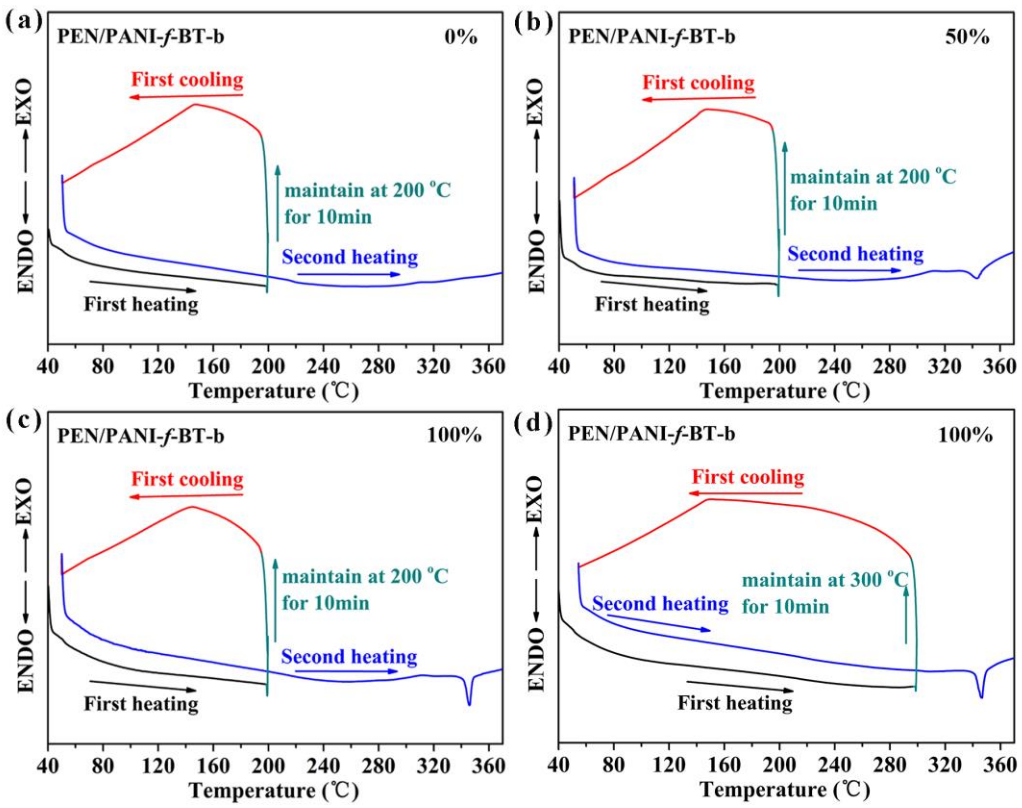

Figure S2. The DSC curves of PEN/PANI-*f*-BT-b nanocomposite films with different stretching ratios: (**a**) PEN/PANI-*f*-BT-b nanocomposites; (**b**) PEN/PANI-*f*-BT-b nanocomposites hot-stretched by 50%; (**c**) PEN/PANI-*f*-BT-b nanocomposites hot-stretched by 100%; (d) PEN/PANI-*f*-BT-b nanocomposites hot-stretched by 100% after treatment at 300 °C.

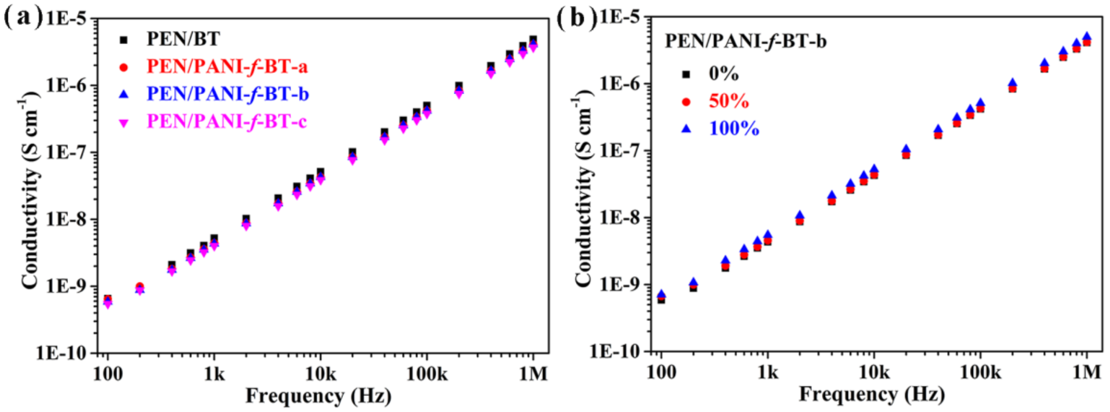

Figure S3. The electrical conductivity of (**a**) PEN/PANI-*f*-BT nanocomposites and (**b**) PEN/PANI-*f*-BT-b nanocomposites with different stretching ratios.
